# Gonorrhœa in the Female*Clinical lecture delivered before class.

**Published:** 1892-12

**Authors:** R. R. Kime

**Affiliations:** Atlanta, Ga., Lecturer on Gynecology in the Southern Medical College


### GONORRHŒA IN THE FEMALE.*

BY R. R. KIME, M. D., ATLANTA, GA.,
Lecturer on Gynecology in the Southern Medical College.

Not long since gonorrhœa in the female was considered of very little consequence, but later, as we learned more of its characteristics, nature and results, the opinion has been reversed. In its direct and remote results it is one of the most serious diseases we have do deal with in the female.

It is a specific contagious disease, usually transmitted by the male ; due to a specific germ or micro-organism called the gonococcus, discovered by Neisser in 1879. It is a diplococcus of different characteristics, microscopically and clinically, from the different varieties of staphylococci and streptococci.

The presence of gonococci in secretions is diagnostic, although their absence in some instances does not disprove it to be the specific element in conveying the disease. It has been demonstrated that the gonococcus has the power to penetrate pavement as well as cylindrical epithelium, and it is also claimed that they can penetrate connective tissue and infect lymphatics. Their presence in the pus of ovarian abscesses and pyosalpinx has been demonstrated, yet they may be absent in these conditions, having run their course, died and disappeared, leaving the pyosalpinx as a result of its ravages.

The source of infection in the female is usually from the

*Clinical lecture delivered before class.

male, yet young girls may contract it from the mother or nurse while caring for them. The course, duration and termination of the disease is influenced by the character of virus introduced, location of infection and characteristics of individual infected. The virus from an acute, active, virulent case is more likely to produce an acute, active, invasive inflammation, running its course more rapidly, especially in subjects with genitalia in condition to form a proper nidus for development of the gonococcus. The virus from a sub-acute, chronic, or what is known as the "latent form," is more likely to run a slower, less active course. In either case, if the patient is of that strumous variety, subject to catarrhal conditions, with genitalia already in an abnormal condition, we may expect more serious results from the infection. Just here I might say he who treats gonorrhœa in the male and fails to warn his patient of the dangerous results in the female of sexual intercourse before he is *completely* cured, or gives consent for marriage before he is thoroughly satisfied that his patient is completely cured, is culpably negligent and derelict of his duty. "The noblest of God's creation, a pure woman," should not be made to suffer for the vices of a man and ignorance of the doctor.

Gonorrhœa may affect the vulvo-vaginal glands, urethra, vagina, uterus, tubes, ovaries, pelvis, peritoneum, and even the rectum. When these different structures become involved, they manifest the usual symptoms of acute inflammation of the several structures. There may be more than one part affected simultaneously, but usually the infection occurs at one point and invades other organs.

It is very difficult sometimes to distinguish a simple vulvitis, vaginitis, urethritis, etc., from specific infection of these organs. In fact, in some cases, the differential diagnosis can only be determined by the presence of the gonococcus. The infection may occur from the male suffering with a latent or chronic form of the disease, in which the virus is deposited high up in the vagina, affecting this point and extending from thence upward without ever involving vulva, urethra, etc. It is thus males often contract gonorrhœa from apparently healthy females, also where the disease is yet lurking in the vulvo-vaginal glands and forced out at sexual intercourse by contraction of bulbo-cavernosa muscles. These militate against the idea of requiring all female prostitutes to be examined to prevent conveying the disease.

The symptoms from this variety of infection may be very misleading as to what would ordinarily occur from gonorrhœal infection. There are such few special manifestations of acute specific inflammation that the disease may be scarcely recognized, and may run its course without hindrance, gradually involving the lining membrane of cervix, uterine body and fallopian tubes, producing pyosalpinx and pelvic peritonitis. It is said the cervix furnishes a favorite abiding place for the gonococcus; that if not molested it will live there for months in luxury, exercising its reproductive functions.

From an infection by virus from the more active acute form we would naturally expect more active inflammation, with more rapid involvement of tissues. Then we would have all the symptoms of acute inflammation manifested in accordance with tissues involved. Thus we might have the characteristics of active acute vulvitis, urethritis, vaginitis, and later endometritis, salpingitis, ovaritis, and even pelvic peritonitis, and as a result of such inflammation, we may have pus accumulate in the fallopian tube (pyosalpinx), or abscess of the ovary.

The cases we have had before the class are instances of a mixed infection according to the latest investigations in bacteriology and pathology. They are instances of gonorrhœal and chancroidal infection, the gonorrhœa having produced a specific vulvitis and vaginitis in each case, and if not controlled by the plan of treatment which we have adopted, later on may develop endometritis and salpingitis, with its consequent results. The chancroidal infection has produced the characteristic chancroids in each case, appearing successively multiple in number, which precludes chancre of syphilis. If multiple, all appear simultaneously. You understand by chancroidal infection that it is a local specific, infectious disease, and not constitutional; that the specific germ has not yet been isolated. This subject will be lectured on later in the course.

In one of our cases we have the rheumatic condition, which is occasionally the result of gonorrhœa, and called gonorrhœal rheumatism. Recognizing the tendency of gonorrhœal inflammation to invade new tissue, you can readily understand how a specific urethritis may develop a gonorrhœal cystitis, which will be lectured on by your able professor of genito-urinary surgery, Dr. Elkin.

We have taken up this subject and dealt with it somewhat collectively from a clinical standpoint. You will be surprised in looking over your gynecological text-books not to find gonorrhœa dealt with to any great extent; not to the extent its importance demands. What you do find will be under the head of local specific inflammation, such as vulvitis, urethritis and vaginitis.

In the treatment of gonorrhœa, briefly considered, we would advise the same general plan for specific urethritis in the female as in the male. For specific vaginitis, thorough cleansing with hot douches, followed with mercuric chloride, 1 to 2,000 or 3,000, well applied; then coat mucous membrane freely with dry boracic acid, and introduce a loose tampon of gauze or absorbent cotton. If preferred, you may apply nitrate of silver, gr. xx to oz. j of water, or comp. tr. iodine and glycerine, equal parts, followed by a tampon of glycerine and boro-glyceride on absorbent cotton. Iodoform gauze may be substituted as a vaginal tampon after either of the above applications. In these cases cleanse cervix well, and close it with iodoform gauze, to prevent extension of disease to cervical and uterine cavity. When it has reached the uterine cavity, dilate cervix with steel dilators (not tents), irrigate at once with mercuric chloride (1 in 2,000), holding cervix open with dilators to allow free return of fluid; then curette to remove coating of albuminate of mercury and dislodge any gonococci buried in uterine lining membrane. After running curette over all the interior of uterus, again irrigate with mercuric chloride, observing same precautions as to escape of the fluid; then tampon with iodoform gauze, with or without an application of carbolic acid, iodine or nitrate of silver to the endometrium. Renew the tampon and applications to endometrium every second or third day until the disease is under control.

If the disease reaches the fallopian tubes, treat as an ordinary salpingitis. If it results in pyosalpinx, which is very likely to occur, then you have the train of symptoms indicative of pus in the tubes, with occasional attacks of local peritonitis. The relief of this condition in the majority of cases will require the removal of the diseased tube. It is advisable in such cases to always remove both tubes unless you are certain one is not diseased, and that you have eradicated all vestige of the disease and gonococci in the uterine mucosa. If the disease has not been entirely cured, all gonococci destroyed, the disease is very liable to extend to the apparently healthy tube and require a second operation. Some of these cases can possibly be relieved by vaginal drainage. It is only advisable, however, when the pus tube is adherent to vagina, and not sacculated, that is, where it has but one cavity with no constricted portions to prevent free drainage. Neither of these cases can be successfully relieved by drainage into uterine cavity. It is best not to temporize with ineffectual measures for relief, but resort to the knife.

